# How Plants Handle Trivalent (+3) Elements

**DOI:** 10.3390/ijms20163984

**Published:** 2019-08-16

**Authors:** Charlotte Poschenrieder, Silvia Busoms, Juan Barceló

**Affiliations:** 1 Plant Physiology Lab., Bioscience Faculty, Universidad Autónoma de Barcelona, 08193 Barcelona, Spain; 2 Plant Sciences, Future Food Beacon of Excellence and the School of Biosciences, University of Nottingham, Leicestershire LE12 5RD, UK

**Keywords:** iron, aluminum, boron, chromium, arsenic, transporter, channel, aquaporin, plasma membrane, vacuole

## Abstract

Plant development and fitness largely depend on the adequate availability of mineral elements in the soil. Most essential nutrients are available and can be membrane transported either as mono or divalent cations or as mono- or divalent anions. Trivalent cations are highly toxic to membranes, and plants have evolved different mechanisms to handle +3 elements in a safe way. The essential functional role of a few metal ions, with the possibility to gain a trivalent state, mainly resides in the ion’s redox activity; examples are iron (Fe) and manganese. Among the required nutrients, the only element with +3 as a unique oxidation state is the non-metal, boron. However, plants also can take up non-essential trivalent elements that occur in biologically relevant concentrations in soils. Examples are, among others, aluminum (Al), chromium (Cr), arsenic (As), and antimony (Sb). Plants have evolved different mechanisms to take up and tolerate these potentially toxic elements. This review considers recent studies describing the transporters, and specific and unspecific channels in different cell compartments and tissues, thereby providing a global vision of trivalent element homeostasis in plants.

## 1. Introduction

Knowledge on inorganic cation transport systems in plants has grown considerably during the last ten years. The combination of molecular-genetic approaches with electrophysiological techniques has allowed us to identify a large number of ion pumps, secondary cation transporters, and specific and unspecific cation channels in different cell compartments and tissues [1,2,3,4,5]. So far, the described cation transport systems refer to monovalent and bivalent cations. Membrane transport rates for trivalent cations are substantially lower than values reported for mono and divalent cations [6], and, apparently, no specific trivalent cation transporters occur in plants. An exception may be NRAMP Aluminum Transporter 1 (NRAT1), a Natural Resistance Associated Macrophage Protein (NRAMP)-type transporter that may function as a specific Al^3+^ transporter (see Section 3.2.1) [7]. Nonetheless, elements with the +3-oxidation state are present inside plant cells and some play essential roles in plant metabolism. After briefly addressing essentiality, the beneficial effects and toxicity of +3-elements in plants, this review will analyze the current knowledge concerning the mechanisms of their uptake and transport. Gaps in our knowledge and future research requirements are highlighted.

## 2. Trivalent (+3) Elements: Essentiality, Beneficial Effects and Toxicity

Besides the Lanthanide and Actinide elements, which all can present the +3-oxidation state, the periodic table of elements further contains 29 elements with the ability to gain a +3-oxidation state. Twenty-five out of these have a metallic character. Under natural conditions, however, only a few of these elements occur in biologically relevant concentrations in soil solutions, sea, or freshwaters. Among those, iron (Fe), manganese (Mn), copper (Cu), molybdenum (Mo), and nickel (Ni) are essential for plants. The functional role of most metal ions with the ability to gain a trivalent state mainly resides in the ion’s ability to change its oxidation state. These ions are involved in the mediation of electron transfer reactions that are vital for chloroplast and mitochondrial functioning (Fe, Mn, Cu) for the antioxidant machinery (Cu, Mn, Fe) and for nitrogen and sulphur acquisition (Fe, Mo) [8]. Nickel is an essential component of urease, the enzyme catalyzing the hydrolysis of urea forming ammonia and bicarbonate [9]. Cobalt has essential functions in prokaryotes, fungi, green algae, animals, and humans. In legume nodules, Co is required for symbiotic nitrogen fixation by diazotrophs and the element is now considered beneficial for plants [10].

Among the essential nutrients, the only element with +3 as a unique oxidation state is the non-metal, boron (B). Its essential function apparently resides in the ability to cross-link plant pectins by forming stable borate esters with cis-diol groups of rhamnogalacturanan-II (RGII) in the cell walls [11,12]. Lewis [13] recently questioned the essentiality of B. By revising the existing experimental evidence, he defends the hypothesis of B being a non-essential, toxic element with indirect positive effects. This author argues that the evident B-deficiency symptoms described in multiple studies are in fact toxic effects of polyphenolic compounds that are not complexed by B under low B supply conditions. This particular point of view should stimulate a new experimental focus to advance the characterization of the biological significance of B in plants. The positive effect of aluminum (Al) on B deficient tea plants provides indirect support to this hypothesis. In B deficient tea roots, Al is mainly bound to cell wall phenolics, thus reducing their availability for enzymatic reactions and lignin biosynthesis [14].

Beneficial effects in plants have occasionally been reported for different elements with either a single +3-oxidation state, such as Al and lanthanum (La), or with multiple valences, including +3, like Cr, Ti, V, and certain rare earth elements [15,16,17,18]. However, there is no evidence for the direct metabolic functions of these elements. Hormesis, mobilization of iron, and multiple other indirect mechanisms may account for their growth promoting effects [19,20,21].

Taking together current knowledge, it is evident that evolution has excluded elements with the single oxidation state +3 from biological functions. Scarce terrestrial abundance as a reason for this exclusion may apply to some elements but is not a plausible general argument. In fact, Al is the third most abundant element in the earth crust. Its availability to plants, however, is limited to acid soils [22]. Either (or both) low bioavailability or high toxicity may be the main reasons for the exclusion of trivalent elements during the evolution of biochemical functions.

According to Exley [23], Al^3+^ has not participated in biochemical evolution because of its scarce bioavailability. The formation of hydroxyaluminosilicate complexes may in part be responsible for this. He argues that enhanced bioavailability of Al is a recent phenomenon related to human activity, which, in combination with decreasing silicon availability, now causes toxicity and forces biochemical evolution to proceed under the pressure of enhanced Al^3+^ bioavailability. Although attractive and certain for areas affected by industrial pollution, this view may not apply to all acid environments. It hardly matches with the fact that Al-hyperaccumulating species have evolved in different botanical families long before the anthropogenic influence. Al-hyperaccumulators are mostly woody plants growing on acid tropical soils with an apparent high bioavailability of Al [24]. Furthermore, Al excluders present specific Al-induced tolerance mechanisms [25]. Their presence implies that Al should have acted as a selection factor during plant evolution. The pleiotropic genetic linkage between Al tolerance and the efficient acquisition of phosphorus [26], an essential macronutrient that is scarcely available in acid tropical soils, may have favored the evolution of Al tolerance mechanisms in these habitats.

The high toxicity of trivalent cations can be a further important factor for the preclusion of these elements from biological functions. Both biological functions and the toxicity mechanisms of chemical elements closely relate to their bioinorganic properties; i.e., their electrostatic interactions and binding characteristics.

Due to their high charge, trivalent cations have a strong influence on the electrochemical properties of cell membranes. Three interacting potentials define these properties: the surface potential (zeta potential or surface charge), the dipole potential, and the transmembrane potential. Trivalent cations can neutralize or even invert the surface charge of negatively charged surfaces. As a consequence, the conductivity of specific voltage-dependent cation channels is altered [27]. The dynamic interactions between the dipole and transmembrane potentials further regulate the transition of the conformational states of voltage-gated channels [28]. The dipole potential arises from the alignment of the dipoles of water molecules and phospholipid head groups. Exposure of pure phosphatidylserine membranes to trivalent cations like the lanthanide gadolinium (Gd^3+^) caused a strong rise of the dipole potential and a six-fold increase in membrane tension. Strong impairment of the functioning of mechano-sensitive channels could be a direct consequence [29]. Lanthanides, especially La^3+^, have widely been used to block Ca^2+^ channels and to study the involvement of Ca^2+^ in signal transduction. However, this La^3+^ action is rather unspecific [30]. The La^3+^-induced impairment of multiple types of plasma membrane channels could be a consequence of the alteration of the membrane’s dipole potential rather than being caused by specific binding to the channel protein.

Metals with +3-oxidation state have a large charge -to- size ratio and typically belong to the hard Lewis acids (type A metals). These ions have relatively low affinity for thiophilic ligands and a low ability to block the essential functional sites of biomolecules with amino or thiol groups or to displace from the enzymes essential transition ions, like Fe^2+^, Zn^2+^, or Cu^2+^ with higher affinity for their ligands. Contrastingly, oxygen donors are the preferred ligands for +3 elements. According to their charge-to-size ratio, the affinity of selected +3 elements follows the series Fe^3+^ < Cr^3+^ < Al^3+^ << B^3+^. Indeed, B^3+^ and Al^3+^ have an extraordinarily high formal charge-to-size ratio and form extremely strong covalent bonds with oxygen. The hydroxyl and carboxyl groups in the polysaccharides and the glycoproteins of cell walls [31,32], phosphate groups [33] in plasma membrane phospholipids [34], nucleic acids, and ATP [35,36] are among the potential binding and toxicity targets for trivalent cations. This binding can have severe consequences for the functioning of cell walls, cell membranes, cell energy budget, cell division, and gene functioning. The described effects imply stiffening of the root cell walls [37,38,39], increased packaging of the membrane lipids, and higher gel to fluid transition temperatures [40]. Conformational changes in the membrane proteins leading to the alteration of ion channel functioning, and signal transduction pathways seem typical primary targets of trivalent cations.

The prooxidant activity is a further important factor of ion toxicity causing oxidative stress by the enhanced production of reactive oxygen species (ROS). Redox-active metals like Fe, Cu, Mn, Cr, Co, Mn, or even Ce, promoting Fenton or Fenton-like reactions, are not the only reactions responsible for oxidative stress induction. Non-redox metals with +3 as the only oxidation state can also trigger ROS formation and lipid peroxidation [41,42,43]. Indeed, in a tobacco cell suspension culture, the metal ion concentration required for superoxide anion production decreased with increasing valence from mono- to di- and trivalent cations [44]. Among the trivalent cations, this oxidative power increases in the order La^3+^ < Y^3+^ < Sc^3+^ < Ga^3+^ = Al^3+^ [45].

Toxicity of trivalent elements causing strong interference with essential plant functions is, most probably, the evolutionary driver for mechanisms that allow plants to handle these elements in a safe way within a certain range of bioavailable concentrations. This is achieved by either or both the exclusion of these elements from the cytosol and the development of mechanisms allowing internal detoxification [46].

After briefly considering Ni, Cu, Co, and Mn trace elements with an unstable +3-oxidation state, this review focuses on Al and B as representative for elements with only the +3-oxidation state, and on Fe, Cr, Mo, As, and Sb for multi-valent elements with a stable +3 state. The different Lanthanide and Actinide elements are not specifically addressed here. Although there is increasing environmental concern regarding these elements [47], the molecular mechanisms behind differential uptake and tolerance by plants are still poorly established.

### 2.1. Elements with Unstable but Biologically Relevant +3 Oxidation States Subsection

The high standard electrode potential (E°) of some multivalent transition elements favors the +2 over the +3-oxidation state (Table 1). Consequently, states such as Ni^3+^, Cu^3+^, Co^3+^, or Mn^3+^ are environmentally unstable and uncommon. The environmental availability of these elements is restricted to the bivalent (Ni^2+^, Co^2+^, Cu^2+^) or monovalent (Cu^+^) states and plants take up these metals by specific or unspecific bivalent cation channels or transporters. However, the +3 state of these elements plays an essential functional role in the metal chelates in diverse biological systems.

#### 2.1.1. Nickel

The hydrated Ni (H_2_O)_6_^2+^ is the main form of Ni in soil solution [50]. Nickel in hyperaccumulating *Odontarrhena* species can be taken up through Ca channels or by an Fe deficiency-induced uptake system [51]. The yellow stripe Fe-nicotianamine transporter protein YSL may transport Ni in the hyperaccumulator *Thlaspi caerulescens* [52]. Nicotianamine (NA) forms stable complexes with FeIII, but Ni(II) has been identified as the species transported by the *Zea mays* ZmYSL1 [53]. Standard electrode potentials can be much lower in complexes, and Ni(III) may be relevant in biological systems. In fact, electron paramagnetic resonance (EPR) studies revealed redox states Ni(III)-Fe(II) in Ni/Fe hydrogenases present in Archae and bacteria [54] where they catalyse the oxidation of H_2_ to H^+^ in a reversible reaction. No such activity seems to be present in plants.

#### 2.1.2. Copper

The main bioavailable form of Cu in soil solution is Cu^2+^ [55]. Under a low Cu supply, Cu^2+^ is reduced to the cuprous ion, and Cu^+^ is the main form taken up by plants with the help of the Cu-specific transporters of the Copper Transporter (COPT) family. Under a high Cu supply, Cu^2+^ is translocated through the tonoplast to vacuoles by Heavy Metal Associated Domain (HMA5) [56]. Divalent Cu chelated by NA is also the oxidation state for long-distance translocation and membrane transport by YSL-transporter proteins [57]. The biochemical relevance of trivalent Cu is under debate. Intermediates in the O_2_ reduction by monooxygenases may imply Cu(III)-peroxide or Cu(III)-hydroxide intermediates [58].

#### 2.1.3. Cobalt

The free Co^2+^ is usually the main species of bioavailable cobalt in acidic soils [59]. This bioavailability decreases with increasing pH. However, the adsorption of Co^2+^ to soil organic matter and iron and manganese oxides also has a strong influence on Co absorption by plants [60]. The membrane transport of cobalt in plants occurs in the form of Co^2+^ by iron transporting proteins [61]. Iron-Regulated Transporter (IRT1), the Fe^2+^ transporter located in the plasma membrane, mediates Co uptake into root cells. The tonoplast-located ferroportin (FPN) transporter FPN2 drives the sequestration of Co^2+^ into root cell vacuoles. Cobalt can also be translocated by FPN1, a plasma membrane transporter located in the stele responsible for the vascular loading of Fe. Under excess Co availability, the cation diffusive facilitator protein MTP1 is implicated in Co tolerance by sequestering Co^2+^ in the vacuoles [62]. Transport of Co^3+^ has not been reported in plants.

Cobalt is part of the corrinoid prosthetic group of cobalamin-dependent enzymes with essential functions in many prokaryotes and eukaryotes, including green algae, but not plants. The corrin cycle stabilizes Co in oxidation states of +1 to +3, and cobalamin-dependent enzymes are involved, among other processes, in electron transfer, methyltransferases, and mutases. The +3-oxidation state of Co also occurs in non-corrin enzymes, such as acetonitrile hydratase from *Rhodococcus sp*. [63]. However, Co^2+^ is the main transport form in organisms with an essential requirement for Co, e.g., through nickel-cobalt transporters of the ATP-binding cassette-type (ABC-type) [64] in bacteria and fungi. Cobalamin-containing CoIII can also enter cells either through receptor-mediated endocytosis, as observed in human cells [65] or as free, non-protein bound cobalamin through ABC-type transporters, as observed in prokaryotes [66].

#### 2.1.4. Manganese

Manganese in soils can present +2, +3, and +4-oxidation states. The plant’s available form is the +2 state, either as Mn^2+^ or frequently complexed with organic compounds. Alkaline pH sharply decreases Mn^2+^ concentrations in the soil solution, while reducing conditions enhance availability. Manganese oxidizing bacteria can produce biogenic insoluble manganese oxides like birnessites (Na_0.3_Ca_0.1_K_0.1_)(Mn^4+^,Mn^3+^)_2_O_4_·1.5 H_2_O or byxbyite-like Mn_2_O_3_ containing Mn in the +3 state [67]. Plants take up Mn only in the +2-oxidation state. Transporter proteins from different families can use Mn^2+^ as a substrate: NRAMP, Yellow Stripe-Like (YSL), IRT and ZIP, Vacuolar Iron Transporter (VIT), Calcium/Proton Exchanger (CAX), Cation/Calcium Exchanger (CCX), Cation Diffusion Facilitator/Metal Tolerance Protein (CDF/MTP), and P-type ATPase [68]. Most transporters are not Mn^2+^ specific and can also transport other divalent or even monovalent (CCX family) cations [68]. Mn^2+^ can activate multiple plant enzymes [69]. Nonetheless, in many of these enzymes Mg^2+^ or other divalent cations can substitute for Mn^2+^. A specific requirement for Mn is found in Mn- Superoxide Dismutase (SOD) responsible for superoxide anion detoxification in the mitochondria and peroxisomes of plants. Mn-SOD contains Mn(III), and, during the dismutation reaction of the superoxide anion (·O_2_^−^) to H_2_O_2_ the Mn(III) is transiently reduced to Mn (II) [70], according to the following equations:

Mn(III) + ·O_2_^−^ ↔ Mn(II) + O_2_ (1)

Mn(II) + ·O_2_^−^ + 2H^+^ ↔ Mn(III) + H_2_O_2_ (2)

The Mn (III) oxidation state, furthermore, plays an essential specific role in the water-splitting complex of the chloroplasts where, under light conditions, electrons are transferred from water to photosystem II and oxygen is evolved:

2 H_2_O → O_2_ + 4H^+^ + 4 e^−^ (3)

**Figure 1:**
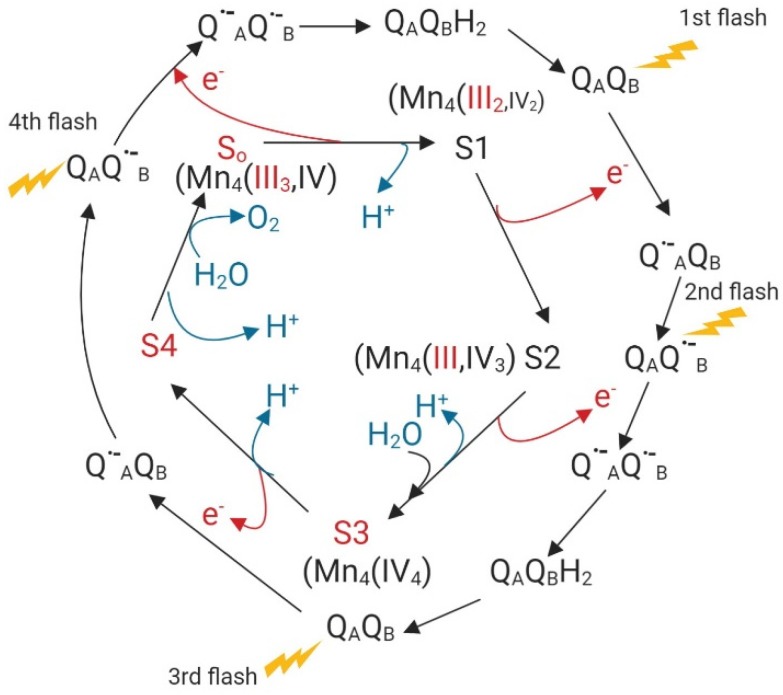
Electron transfer from water to Q_A_Q_B_, the acceptor site of photosystem II (PSII) based on Kok’s clock, modified with permission from [71]; published by Springer Nature, 2018. Red arrows, electron transfer; blue arrows proton release.

Based on Kok’s clock, the transference of four electrons from water to the acceptor site at photosystem II (PS II) implies four different states in which Mn (III) and Mn (IV) facilitate electron transfer. Figure 1 illustrates the different oxidation states of Mn in this electron transport process [71].

## 3. Elements with Only a +3 Oxidation State: Boron and Aluminum

Among the elements with a single oxidation state of +3, B and Al have found the most research interest among plant scientists. Aluminum and B share group 13 in the periodic table of elements, displaying 3 electrons in their outer shell. Due to its small ionic radius and high ionization energy (3rd ionization enthalpy 3660 kJ·mol^−1^), B does not form B^3+^ salts. Further, the hydrated B^3+^ (aq) is unknown. Boron is found in nature in the form of borax Na_2_B_4_O_7_·10H_2_O and as neutral boric acid H_3_BO_3_ that may hydrolyze to the borate anion B(OH)_4_^−^ under alkaline conditions (pK 9.2). With increasing soil pH, the adsorption of B to Al and Fe oxides, Mg hydroxides, clay minerals, and organic matter increases. It decreases again in highly alkaline media [72].

Aluminum with a larger ion radius and lower third ionization energy (2745 kJ·mol^−1^) than B is stable under acidic conditions (pH < 5) in a cationic form as a hexahydrate Al(OH)_6_^3+^, usually abbreviated as Al^3+^. This trivalent cationic Al^3+^ is the main phytotoxic form in acid mineral soils. Under increasing soil pH, progressive deprotonation gives rise to the less toxic Al(OH)^2+^ and Al(OH)_2_^+^. The sudden alkalinization of acid solutions containing Al^3+^ can foster the formation of Al_13_. This polymeric Al species is highly toxic to plants. However, its presence under natural soil conditions is uncommon [73].

Sparingly soluble Al-silicate, Al-phosphate, and Al-polyphosphate complexes are non-toxic. Low toxicity is also observed for Al-sulphate, Al-fluoride, and Al-complexes with low molecular weight, as well as for soluble organic compounds, like organic acids or phenolics [46,74].

Although Al and B differ in their metallic and non-metallic characteristics, they apparently share common binding properties that promote strong interactions between both elements within plant tissues (see Section 3.2.2).

### 3.1. Boron

The fact that under low B supply plants can show severe deficiency symptoms and yield reductions has stimulated studies into the uptake, transport, and functions of B in plants over the last 80 years [75]. Moreover, B toxicity responses and tolerance mechanisms are of interest, especially considering the narrow ranges of optimal B concentrations that may considerably differ among diverse crops [76]. As a consequence, B concentrations, which are optimal for plants with low requirements (e.g., cereals) may be deficient for more exacting crops, such as cabbage or asparagus [77]. Contrastingly, B toxicity can occur in low B species when cropped either after high demanding species that have received the fertilizer supply or in soils with a high B availability due to either natural or anthropogenic causes [78].

Boric acid or H_3_BO_3_ (also written as B(OH)_3_) is the main B species taken up by plants. In this neutral form, B may diffuse through the plasma membrane. Artificial lipid membranes are quite permeable to boric acid, with permeability coefficients in the range of 4.9·10^−6^ cm·s^−1^ to 9.5·10^−9^ cm·s^−1^ depending on pH, the presence of cholesterol, the type of lipid head groups, and the chain length of the fatty acids. Experiments using *A. thaliana* mutants differing in plasma membrane composition further demonstrated the strong influence of membrane lipid composition on the B uptake [79]. Studies on the giant cells of *Chara corallina* revealed two B uptake systems. One for a low B concentration saturating at 5 µM (corresponding to facilitated transport) and a second, linear, phase ascribed to simple diffusion [80].

Nodulin26-like Intrinsic Proteins (NIPs) facilitate the diffusion of boric acid across membranes [81]. NIPs are channels of the aquaporin family. NIP channels can transport uncharged hydroxylated forms of other +3-elements, such as Sb(OH)_3_ and As(OH)_3_ [82], or +4-elements in an uncharged form, like silicic acid, H_4_SiO_4_ [83], and selenous acid, H_2_SeO_3_ [84]. The transport specificity of aquaporins is apparently determined by two constriction regions: one located in the channel center formed by two NPA (asparagine-proline-alanine) motifs and a second selectivity filter located extracellularly at the mouth of the channel formed by an aromatic/arginine region (ar/R). The ar/R selective filter seems to be of primary importance for B-transport activity [85]. Variability in this region is responsible for the broad spectrum of small molecules transported by the aquaporin channel family [86]. *A. thaliana* NIP5;1, which is permeable to both water and boric acid, is located in the plasma membrane of the root epidermal cortex and endodermal cells [81]. Contrastingly, NIP6;1, which is mainly expressed in stems, is impermeable to water and is responsible for distribution of B in the growing shoot tissues [87]. NIP7;1, with strong expression in floral tissues, seems to favor B-transport to the developing tapetal tissue of developing anthers [88].

**Figure 2:**
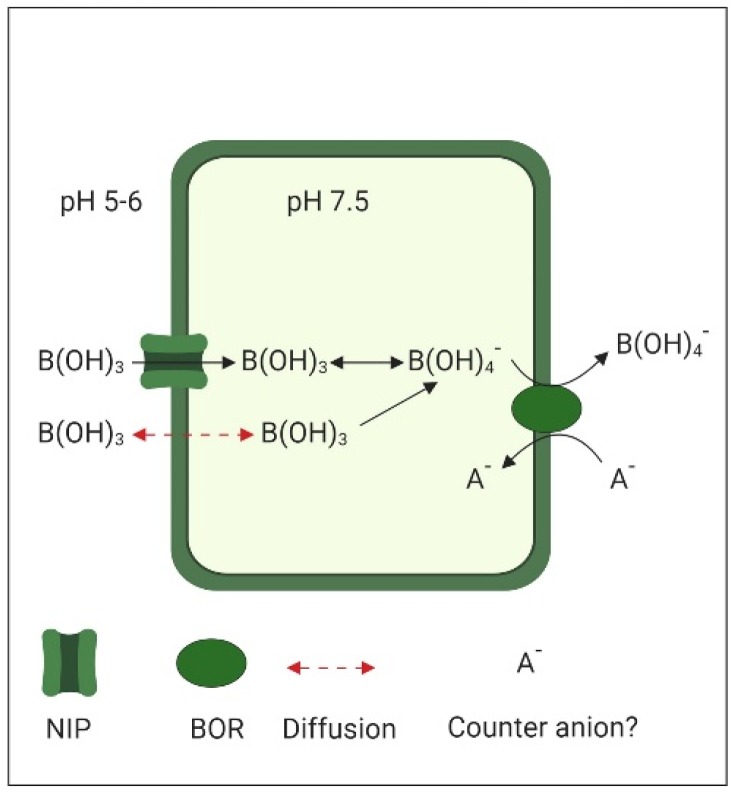
Boron (BOR) transport in plant cells. Neutral boric acid, the main form in the slightly acidic apoplast, can enter either by diffusion or through the Nodulin26-like Intrinsic Protein (NIP). The near to neutral pH of the cytoplasm favors the formation of borate, which is exported by BOR transporters.

While neutral boric acid B(OH)_3_ is the main form of B in the slightly acidic apoplastic space (pH 5 to 6), once it has crossed the plasma membrane, inside the nearly neutral cytoplasm (pH 7.5), the formation of B(OH)_4_^−^ is favored (Figure 2). This anionic borate is the substrate for the BOR1 (Boron transporter 1), a membrane transport protein of the Slow Anion Channel-associated 4 (SLAC4) family known as bicarbonate transporters, due to their homology to the human bicarbonate transporter Band 3. SLAC4-type transporters operate either by electroneutral anion exchange or by sodium–driven anion exchange [89]. Detailed structural characterization indicates that BOR1, like other members of the SLAC4 family, works by alternating access to both sites of the plasma membrane using an elevator mechanism [90]. The exchanged counterion used by BOR1 is still unknown.

NIP5;1 and BOR1 present an opposite polar distribution in the root cells of *A. thaliana*. While the influx channel NIP5;1 is located in the outer polar domain of the plasma membrane of root cells, BOR1 occupies the inner domain. This allows the efficient radial transport of B from the soil through the epidermis, cortex, and endodermis to the stele [91]. In addition, BOR2 seems to be involved in the efflux of B from the symplast to the apoplast favoring B cross-linking of rhamnogalacuronan II in the root cell walls and supporting root cell elongation [92]. Clathrin–mediated endocytosis (CME), dependent on AP2 (Adaptor Protein 2) and mediated by Dynamine-Related Protein A1 (DRAP1), is required for the maintenance of the polar localization of these B-transporters [93].

Genes coding for B-transporters and channels are considerably upregulated under a B-deficiency. Contrastingly, under a high B supply, the fast degradation of BOR1 in the vacuole regulates the restriction of excess B transport to the shoots. This is achieved by fast CME and vesicle transport from the inner plasma membrane domains to the vacuole [89]. This B-induced vacuolar sorting is AP2-independent [94]. The tissue specific expression of NIP and BOR1 transporters is highly sensitive to the B nutritional status of these tissues. Thus, a fine regulation of this expression is fundamental for the maintenance of B homeostasis under both deficient and excessive B supply [95].

### 3.2. Aluminium (Al)

There are two main reasons for the boost of the research interest on Al in plants during the last years. One is the progressive acidification of surface waters and soils due to acid rain, causing enhanced bioavailability and toxicity of Al. The other refers to the intent to put large areas of acid tropical soils with naturally high Al saturation under intensive agriculture [74,96]. Both mechanisms of Al phytotoxicity and, especially, the genetic and molecular basis of the differential plant tolerance to high Al availability have focused these research efforts [25].

#### 3.2.1. Phytotoxicity of Al

Al^3+^ is the main phytotoxic Al species [97] that causes severe inhibition of root growth within minutes upon exposure. The Al-sensitive root zone is the distal transition zone where cells initiate fast expansion [98]. The time required for detecting the toxic Al effects on root or root cell elongation varies from 5 to 30 minutes, depending on the Al concentration and the plant species [39,99,100]. Direct cross-linking by Al^3+^ of the negatively charged pectin carboxyl groups in the cell walls and/or Al-induced production of active oxygen species may be responsible for cell wall stiffening and such a fast inhibition of cell elongation [39,101]. Coincidence in the time of the Al-induced inhibition of both root elongation and pectin recycling in root tips supports the role of Al binding to cell wall pectins as a fast, and initially reversible, mechanism of Al-induced root growth inhibition [102].

Conspicuous alterations of endogenous root hormone levels occur within the timespan required for Al-induced root growth inhibition. A 60% to 80% decline of cytokinin nucleotides, a six-fold increase in zeatin levels after 5 min, and a transient increase of ethylene after 15 min occur in roots of bean seedlings exposed to 50 µM Al [103]. The aluminum-induced inhibition of basipetal auxin transport was observed within 30 min upon receiving the Al-supply [104]. These results are in line with a fast Al-induced alteration of signal transduction pathways, leading to the alteration of root tip cell patterning [105]. This may be a consequence of Al’s interaction with the cell wall properties or its interaction with the root tip plasma membrane, or both.

#### 3.2.2. Al Uptake, Transport, and Tolerance

Exclusion of Al from the sensitive root tips is a key feature of Al tolerance in plants. However, there is now increasing evidence that mechanisms of internal detoxification also play a relevant role. Plant species largely differ in their Al tissue concentrations. Most species preferentially accumulate Al in their root tissues and restrict Al access to the leaves. When exposed to Al under acidic conditions the increased shoot Al concentrations usually do not exceed a few hundred mg·kg^−1^ dry weight. Contrastingly, accumulator or hyperaccumulator species have leaf Al concentrations ranging from 1000 to more than 10,000 mg·kg^−1^ leaf dry weight [24,106]. These species from different botanical families are almost exclusively woody shrubs or trees growing on tropical soils, including some cropped species like the tea plant [107], buckwheat [108], and hydrangea [109]. Aluminum (up to several thousands of mg·kg^−1^ dry weight) also accumulates in the leaves of hemi-parasitic mistletoes feeding on the xylem tissues of Al hyperaccumulating tree species [110].

Expectedly, differences in Al transport systems seem to be responsible for large differences in Al uptake and transport rates among species. However, our knowledge on the molecular basis of Al transporters is just emerging. The complex, pH-dependent speciation chemistry of Al hampers investigations into the uptake and transport mechanisms of Al in plants. Moreover, the high affinity of plant cell walls leading to preferential, massive Al accumulation in the apoplast may mask symplastic Al (see [111] for a detailed discussion of technical difficulties). Studies with *Chara corallina* after removing the cell walls [112] clearly revealed the uptake of ^26^Al through the plasma membrane and the tonoplast of the protoplasts of the giant cells of this algal species. This uptake was linear for several hours before saturation. Secondary ion mass spectrometry revealed Al entrance into the root tip cells of soya beans within 30 minutes [113]. Aluminum accumulated in the vacuoles of an Al-tolerant maize variety within 4 hours upon exposure. Roots of these plants revealed a low apoplastic accumulation of Al due to Al-induced organic acid exudation [114].

Exley and Mold [115] proposed four hypothetic ways for Al to cross the plasma membrane in biological systems: by diffusion through the lipid bilayer of neutral, low molecular weight organic Al complexes, by active transport of anionic or cationic organic Al complexes, through channels, or by adsorptive or receptor-mediated endocytosis for organic Al complexes or micro- or nano-particulate Al. They further envisaged a paracellular pathway for Al^3+^ between cells for animal or human epithelic and endothelic tissues. In plant tissues, this would correspond to the apoplastic space.

Recently, experiments using molecular-genetic approaches provided evidence for the Al membrane transporter systems in plants. ART1 is a transcription factor for Al tolerance in rice [116], regulating 32 genes. One of these genes encodes NRAT1, a protein of the NRAMP divalent cation transporter family. NRAT localizes in the plasma membrane of the epidermal and outer cortex cells. Heterologous expression in yeast shows specific induction by Al^3+^ and not by La^3+^, low pH, or bivalent cations. The knockout lines of NRAT1 show lower root cell sap Al and higher cell wall Al concentrations than the wild type, while no influence on the concentrations of other mineral elements was found. These results suggest that NRAT1 may function as a specific Al^3+^ transporter in rice [117]. However, it should be taken into account that at pH 4.2 the presence of monomeric Al(OH)^2+^ or Al(OH)_2_^+^ in the culture medium cannot be precluded.

**Figure 3:**
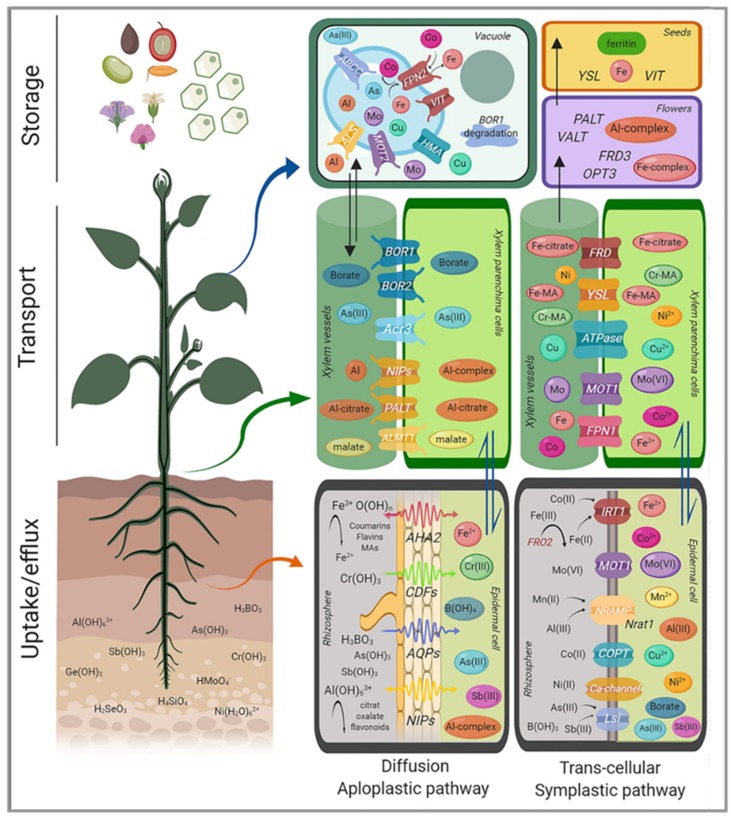
Overview of trivalent elements homeostasis in plants. Uptake systems and main transporters involved in loading, efflux, and storage are indicated for each element: Aluminum (orange), Arsenite (light blue), Boron (dark blue), Cobalt (pink), Copper (turquoise), Chromium (green), Iron (red), Manganese (light yellow), Molybdenum (purple), Nickel (dark yellow), Antimony (violet). AHA, H+-ATPase; CDFs = Cation diffusion facilitators; AQPs = Aquaporins; LSI, silicon transporter; Mas = Mugineic Acids; NIPs = Noduline Intrinsic Proteins.

The electroneutral Al-malate, but not Al^3+^, is transported through the plasma membranes of *A. thaliana* by NIP1;2 [118] (Figure 3). This bidirectional transporter of the NIP channel family (see Section 3.1) removes Al from the root cell wall and favors the entrance of Al-malate into the cytoplasm. This mechanism of Al internalization to the cytoplasm depends on Al-activated malate efflux from the roots, which is mediated by AtALMT1 [118], a malate efflux carrier that is essential for the detoxification of Al in the apoplast of the Al sensitive root tips of *A. thaliana*. Inside the root cells, Al-malate can be compartmentalized into the vacuole. AtALS3 and OsALS1 have been identified as vacuolar Al transporters in *Arabidopsis* and rice, respectively [119]. Export to the xylem can be achieved by NIP1;2 [120]. Besides malate, other organic acids released by roots of different species can form non-toxic, stable complexes with Al. Examples are, among others, oxalate exudation in buckwheat and tea, or citrate in maize and sorghum [46]. Further, phenolic compounds, especially flavonoids can tightly bind Al in the apoplast and avoid the rhizotoxicity of Al [121]. Aluminum-induced organic acid efflux can be triggered immediately upon Al exposure (pattern 1) or after a lag time of several hours (pattern 2) [122]. Whether Al–organic acid complexes other than Al-malate can cross the plasma membrane from the cell wall to the cytoplasm using specific plasma membrane transporters is still not clearly established. In the Al-accumulating *Hydrangea macrophylla*, high Al accumulation in the sepals is responsible for the development of a blue color. Sepal-specific expression of the plasma-membrane located in HmPALT1 (along with HmVALT1, located in the tonoplast) seems responsible for this tissue-specific Al accumulation [123]. It is unclear what Al species are transported by these proteins. Al-citrate is the main Al species in the xylem sap of *H. macrophylla* [124]. HmPALT2 has been proposed as an Al-citrate transporter responsible for the long-distance transport in *H. macrophyla* [125]. This anion permease of the ArsB/NhaD family is expressed throughout the plant and may transport other metal or metalloids, as well as anions.

Boron and Al located, respectively, in the first and second periods of group 13 of the periodic table show strong interactions within plant tissues. Both elements can cross cell membranes using proteins of the aquaporin family. This suggests a possible competition on the transporter level. In fact, surplus B ameliorates Al toxicity in different species [126,127]. In turn, Al has a positive effect on B-deficient tea plants [14]. In rice, B-pretreatment reduces Al-binding to the cell wall pectins and increases the expression of the OsSTAR1/STAR2 involved in reducing the accumulation of Al in cell walls. Boron pretreatment also enhances the expression of OsALS, facilitating Al storage in the vacuole [128]. The possible interaction of Al and B in NIP transporters clearly deserves further exploration.

Positive interactions between B and Al may also be related to a decrease in oxidative stress. Boron enhances antioxidant defenses in Al-stressed plants [128,129]. Aluminum, in turn, may improve the performance of B-deficient plants by binding excess polyphenols in the cell wall [14].

## 4. Polyvalent Elements with a Stable +3-Oxidation State: Iron, Chromium, Molybdenum, Arsenic, and Antimony

### 4.1. Iron

Iron is essential for all organisms where it is involved in fundamental electron transport processes, as a cofactor in iron sulphur clusters, and in heme proteins, among others [130]. The redox properties that make Fe biologically indispensable imply that the free Fe ions, Fe^3+^ or Fe^2+^, are highly destructive. The reduction of Fe^3+^ to Fe^2+^ and its oxidation by H_2_O_2_ (Fenton reaction) yield the hydroxyl radical (·OH) or an oxidoiron (IV), which are both highly oxidizing and toxic species. Consequently, Fe uptake must be highly regulated, and Fe metabolism is highly compartmentalized [131].

#### 4.1.1. Iron Uptake

While the abundance of Fe in soils and waters is relatively high, Fe availability is scarce under aerobic conditions due to the fast formation of Fe(III) oxides and hydroxides with low solubility [132]. The concentrations of free Fe^2+^ and Fe^3+^ in soil solution are extremely low. Soluble Fe is mainly found in the form of organic complexes with organic acids, phenolics, and humic substances [133,134].

As most of the Fe in soil is present in poorly soluble forms, plants have to mobilize Fe before uptake. Two main approaches can be distinguished. Strategy I, which seems to be the most ancient mechanism (since it is used by dicots and some monocots), and Strategy II, which is carried out by all monocot grasses [135].

In Strategy I, Fe is mobilized through acidification by the activity of proton pumps (H^+^-ATPase), particularly H^+^-ATPase 2 (AHA2) (Figure 3) [136] and via chelation by low molecular weight compounds, such as organic acids and coumarins, which are secreted into the rhizosphere [137,138]. The mobilized ferric Fe(III) diffuses into the apoplast, followed by the reduction of Fe(III) to ferrous Fe(II) via plasma membrane-bound ferric chelate reductase enzymes [139] and coumarins [140,141]. In *A. thaliana*, rhizosphere Fe(III) is reduced by Ferric Reductase Oxidase 2 (FRO2) [139] and then transported as Fe^2+^ into the epidermal cells by the divalent metal transporter Iron-Regulated Transporter 1 (IRT1) [142], which also transports Zn^2+^, Mn^2+^, Cd^2+^, Co^2+^ [143], and Ni^2+^ [53].

This tightly controlled process prevents Fe overload of the plant using at least three levels of regulation: transcriptional, posttranslational, and intracellular trafficking [144,145]. At the transcriptional level, a number of basic helix loop helix (bHLH) factors control the expression of these genes [146,147]. Ubiquitinization plays a role in controlling the protein levels of some of these transcription factors, as well as the membrane recycling of IRT1 [148].

Barley, rice, and maize in the grass family (Poaceae) represent Strategy II plants, which secrete phytosiderophores, defined as plant-derived small organic molecules with a high affinity for Fe. In Strategy II, iron is not membrane transported as Fe^2+^, but as an Fe(III) complex with mugineic acids (MA), a NA derivative with high affinity for Fe^3+^ (Figure 3) [145]. Subsequently, the complex is introduced into the plant by YSL transporters, first characterized in maize [149], and later in rice (YSL15) [150]. For transport to occur, MAs must be extruded to the rhizosphere mediated by the transport of mugineic acid TOM1-like transporters [151]. This process is also under transcriptional control, with iron deficiency-responsive cis-acting elements (IDEF1, IDEF2) and the iron-related transcription factor 2 (IRO2) being some of the key transcription factors involved [152,153,154].

The separation of these two strategies is not as simple as originally thought. Many Strategy I plants release, in their root exudates, a number of molecules that can solubilize Fe; some of them even can reduce Fe(III) to Fe(II). These include phenolics, coumarin carboxylates, and flavins [155]. Phenolics help with the solubilization and reutilization of apoplastic Fe, as initially shown in red clover [156]. Coumarins seem to play a crucial role for Fe acquisition in *A. thaliana* under high pH conditions [137,157,158]. Differential adaptation to calcareous soils in this species is related to enhanced coumarin exudation [159]. Coumarins are synthesized using precursors from the phenylpropanoid pathway. The first coumarin in the pathway, scopoletin, is synthesized by the enzyme feruloyl CoA ortho-hydroxylase 1 (F6′H1) [160]. While there is some uncertainty about the precise biosynthetic steps, the active end-product is most likely fraxetin [140]. Other plant species, such as *Medicago truncatula* and sugar beet, secrete flavins instead of coumarins, which also function to facilitate the reduction of ferric iron [140,161]. In addition, secretion of the polyamine compound putrescine appears to improve the mobilization of iron inside the plant cell wall [162].

#### 4.1.2. Iron Transport

Most Fe enters the plant via the root and is then transported to the sink tissues where it is required for iron-dependent enzymes. IRT1 is predominantly localized in the outward facing membrane of epidermal cells, suggesting that Fe first enters the symplastic pathway here and then moves through the root cortex to the stele by the transcellular pathway using the plasmodesmata cell connections [163]. Besides IRT1, NRAMP1, other low-affinity plasma membrane transporters can also possibly contribute to Fe transport [164].

Iron likely moves symplastically to the pericycle, where it then needs to be effluxed into the xylem to move to the shoot [165]. Ferroportin (FPN) is the sole Fe efflux transporter identified to date in animals, and there are two closely related orthologs in *A. thaliana*, IREG1/FPN1 and IREG2/FPN2. FPN1 localizes to the plasma membrane and is expressed in the stele, suggesting a role in vascular loading; FPN2 localizes to the vacuole and is expressed in the two outermost layers of the root in response to iron deficiency, suggesting a role in buffering metal influx [61].

Metal binding by low molecular weight soluble organic molecules is essential to avoid iron precipitation [166]. Fe(III)-citrate seems to be the form of Fe for long distance transport in the xylem sap [167]. Further support for the preeminent role of citrate as an Fe carrier in xylem sap is the characterization of the Ferric Reductase Defective (*FRD3*) gene in *Arabidopsis* and its orthologue *FRDL1* in rice [168,169]. *FRD3* mutation causes Fe deficiency in shoots and an accumulation of this metal in the roots. This process is consistent with a strong reduction of the root to shoot transport of Fe in the mutant. FRD3 is not only involved in xylem loading of Fe-citrate, but more generally in Fe transport across symplastically disconnected tissues (Figure 3) [170].

In plant leaves, Fe re-enters the symplast and is reduced to Fe^2+^, the biologically active form, mainly by the action of FRO proteins. A large proportion of Fe is used in the plastids, and mitochondria and Fe transporters specific for each type of organelle have been identified (see reviews in [171,172]). Some Fe is remobilized from leaf tissues and reaches other sink organs through the phloem. In *Arabidopsis*, the oligopeptide transporter family protein OPT3 is involved in this process [173].

#### 4.1.3. Iron Storage

Seeds are the last destination of Fe transport in plants. The reserves of Fe stored in seeds are important during germination, before the seedling has developed a functional root system and is able to mobilize and take up Fe from the soil. YSL transporters are involved in seed loading [174], and there is evidence that Fe can be delivered to pea embryos as a Fe(III)-citrate/malate complex [175]. Iron, delivered as iron-citrate with the help of FRD3, is also essential for pollen development [170]. FRD3 could also play a role in embryogenesis, as citrate would solubilize the Fe in the nutrient solution around the developing embryo [170]. However, some other transporters ought to be involved, since no developmental alteration was observed in frd3 embryos. Iron delivered to the embryos is directed to vascular tissues where it is stored in the vacuoles of endodermal cells [176].

Two major storage mechanisms for Fe are proposed: sequestration into vacuoles and into ferritin. In *A. thaliana*, AtVIT1 seems to mediate Fe transport into the vacuoles [177]. The action of the efflux transporters NRAMP3 and NRAMP4 releases Fe into the cytosol during germination [178]. A suppressor screen of nramp3/nramp4 mutants identified mutations in VIT1 that rescue their sensitivity to low Fe [179]. Genes from the VIT-family are also important for Fe localization in rice grains and Brassica seeds (Figure 3) [162,180].

Ferritins are essential iron storage proteins present across the biological kingdoms [163]. The proportion of total Fe stored in ferritin in seeds varies among species, with approximately 60% in peas, but less than 5% in *Arabidopsis* seeds [181]. In plants, ferritin is predominantly located in the plastids. In cereal grains, such as wheat and rice, most Fe is present in vacuoles in the aleurone layer. As this layer is often removed during grain processing, refined food products can have a considerably lower Fe concentration, thus affecting their nutritional value for consumers [182].

### 4.2. Chromium and Molybdenum

Chromium and Mo are transition elements that share group 6 of the periodic table. Molybdenum presents oxidation states from −2 to +6. The most stable and abundant state of Mo in soils and water is +6, where it can be found in soluble forms as MoO_4_^2^^−^ or HMoO_4_^−^. It is also adsorbed onto particles of clay, metal oxides (Fe, Al, or Mn), and organic matter [183]. Plants take up Mo (VI) in its anionic form. Two transporters of the sulphate transporter family have been related to Mo transport in *A. thaliana*, Molybdenum/Sulphate Transporter MOT1/SULT5.2 and MOT2/SULTR51. MOT2 seems to be located in the tonoplast, favoring export of molybdate out of the vacuole under Fe deficient conditions [184]. The location of MOT1 is still uncertain. The essential role of Mo relies on the ability to form Molybdenum Cofactor MOCO or FeMOCO clusters implied in redox processes changing the oxidation states from Mo(IV) to Mo(V) and Mo(VI) [184]. The reduction to Mo(III) needs strong reductants, and, in aqueous solutions, Mo(III) is easily oxidized to Mo(V) and Mo(VI). The trivalent oxidation state of Mo does not seem to play any role in its biochemical redox function.

Chromium can also adopt all oxidation states from −2 to +6. However, in contrast to Mo, not only state +6 but also +2 and +3 are stable. The environmental significance is limited to states +3 and +6. In soils and waters, Cr(III) is the main form, while oxidation yields highly toxic, carcinogenic Cr(VI).

For many years. Cr in its +3 state was considered an essential nutrient for animal and human life. This essential function was ascribed to the presence of Cr(III) in the so-called glucose tolerance factor and the observations of the beneficial effects of Cr(III) dietary supplements on diabetes and lipid metabolism [185]. As unambiguous experimental support is missing and the mechanisms of this supposed essential action have not been clearly established, the element has now been dropped from the list of essential elements for humans and animals [186]. In plants, Cr never has been considered as an essential nutrient, although beneficial effects have been observed, especially under imbalanced nutrient supply [187].

Uptake mechanisms of Cr by plants are still not clearly established. Plants may take up Cr(VI) in the form of chromate by anionic transporters. However, under most environmental conditions, Cr(VI) is readily reduced to Cr(III). This Cr(III) may diffuse into the apoplast quite easily and accumulate in the cell walls by binding to the O-donor groups of the cell wall components. Transport across the plasma membrane into the symplast seems to be slow. In contrast to FeIII, which is enzymatically reduced to Fe^2+^ in roots of dicots (see Section 4.1.1) before crossing the plasma membrane, no reduction of Cr(III) to Cr(II) has been reported for plant roots. The standard reduction potential of Cr(III)/Cr(II) is far too low (Table 1) for the reduction activity at the root plasma membrane surface [188]. Chelation by phytosiderophores and subsequent uptake through YLS transporters could be an option in grasses. In fact, the solubility of Cr(III) is enhanced [189] in solutions spiked with either ethylene diamine tetracetic acid (EDTA) or mugineic acid (MA), a natural phytosiderophore exudated by grasses with Fe-deficiency. However, in contrast to Fe(III)-MA, the Cr(III)-MA complex is apparently not transported through the plasma membrane by YSL transporters in *Zea mays*. This is supported by the lack of differences in tissue accumulation of Cr(III) between the ys1 mutant, which is defective in metal-siderophore uptake and the wild type [189]. Phytosiderophore-mediated Cr(III) uptake has been proposed as responsible mechanism for the huge uptake of Cr(III) in the grass *Leersia hexandra*. Unusually, an about eight-time higher leaf Cr concentration (> 4000 mg·kg^−1^) accumulated in the leaves of this species when exposed to Cr(III) than under a Cr(VI) supply. Cr(III) uptake was inhibited by Fe(III) and it was claimed that phytosiderophore-mediated transport may be responsible for this large Cr(III) accumulation [190]. However, direct experimental evidence for this is process is currently missing. In most plants, Cr translocation from roots to shoots is severely restricted. Even in serpentinophytes growing on ultramafic soils with naturally high Cr concentrations, shoot Cr usually remains below 100 mg·kg^−1^ dry weight [191]. Although species differences in shoot accumulation among serpentinophytes occur, and values close to 500 mg Cr per kg dry weight have been reported in *Euphorbia selloi* and *Drosera montana* [192]. The low shoot translocation of Cr in most plants seems related to a preferential storage of Cr in the root apoplast and the root vacuoles [193,194]. How Cr enters into the vacuole is not established.

### 4.3. Arsenic and Antimony

Arsenic and Sb share group 15 of the periodic table of elements. Both present two biologically relevant oxidation states, trivalent arsenite As(III) or antimonite Sb(III) and pentavalent arsenate As(V) or antimoniate Sb(V). No essential functions have been described for either.

Both As(III) and Sb(III) are taken up into cells of *Escherichia coli* by the glycerol channel GlpF, a member of an aquaporin (AQP) superfamily [195]. In neutral solutions, arsenite As(OH)_3_, an inorganic polyol similar to glycerol, is formed. Aquaglyceroporins seem to be a universal route for As(III) uptake in plants [196]. The plant MIP superfamily falls into numerous subfamilies. Members of the Nodulin 26-like Intrinsic Protein (NIP) subfamily are responsible for facilitating As(III) uptake into plant roots. In *A. thaliana*, at least five NIPs, including NIP1;1, NIP1;2, NIP5;1, NIP6;1, and NIP7;1, facilitate As(III) uptake into roots. NIP3;1 is also involved in the movement of As(III) into the root, and from there, into the shoot [197]. In addition to As(OH)_3_, plant aquaglyceroporins facilitate uptake of other metalloids, including B(OH)_3_, Si(OH)_4_, Sb(OH)_3_, and Ge(OH)_3_. Low Silicon 1 (LSI1) mediates transport of a range of small neutral molecules, including Sb(III), urea, B(OH)_3_, and As(III). In rice roots, LSI1 is highly expressed in the distal side of the endodermal and exodermal plasma membranes where Casparian strips are formed, and LSI1 is a major entry route for silicic acid and As(III) [198]. Mutations in LSI1 resulted in a 60% loss of As(III) uptake. LSI1 also mediates the uptake of undissociated pentavalent methylarsenate (MAsV) and dimethylarsenate (DMAsV). Mutants lost 80% and 50% of the uptake capacity for MAs and DMAs, respectively, compared to wild-type rice [199]. The compartmentation of phyotochelatin-bound As(III) into the vacuoles by ABCC-like transporters is crucial for As tolerance in *A. thaliana* and rice [200,201].

The extrusion of As(III) allows cells to grow in the presence of high external As(III) concentrations. ArsB was the first identified As(III) efflux system and is employed by many bacteria for As tolerance, followed by Arsenical Resistance Protein (ACR3). An ArsB homolog exists in plants and is responsible for the transcellular translocation of As(III) [202]. Some plants also have proteins related to ACR3 that confer As tolerance. For example, an ACR3 ortholog was identified in the vacuole of *Pteris vittata*, an As hyperaccumulating fern, where it is responsible for the extraordinarily high As tolerance of this species [203]. In contrast, rice does not have *ACR3* [198]. However, transgenic rice plants expressing the *S. cerevisiae ACR3* gene increased As(III) efflux and also lowered As accumulation in the grains [204]. The expression of PvACR3;1 from *P. vittata* in *A. thaliana* and tobacco (*Nicotiana tabacum*) was shown to increase As retention in the roots, thereby decreasing As accumulation in the shoots [205]. Recent gene expression studies revealed a close cooperation of different uptake and storage mechanisms responsible for As hyperaccumulation and hypertolerance in the gametophytes of *P. vittata* [206]. Based on a similar mechanism previously established for As tolerance in *Pseudomonas aeruginosa* [207], Cai et al., [206] proposed the following model for As hyperaccumulation and tolerance in *P. vittata*: As(III) crosses the plasma membrane by the aquaporin AsTIP4 and is transported through the tonoplast for vacuolar storage by PvACR3. As(V) enters the cell by the phosphate transporter PvPht1. In the cytoplasm, As(V) is either directly reduced to As(III) by arsenate reductase (PvACR2) or used—instead of phosphate—as a substrate for phosphoglyceraldehyde dehydrogenase (PvGAPC1) to convert glyceraldehyde 3-phosphate to 1-arseno-3-phosphoglycerate. This As(V)-compound is then transferred to membrane-bound vesicles by Organic Cation Transporter 4 (PvOCT4) and reduced to AS(III) by PvACR2 or PvGSTF1. As(III) is delivered to the vacuole by membrane fusion with the tonoplast.

Considerably less information is available for Sb. Antimony and As have common properties but differ in their metallic and non-metallic characteristics, respectively. In its trivalent form, Sb(III) and As(III) seem to share the same transport systems based on aquaglyceroporins [82]. Sb(III) is readily accumulated in roots of *P. vittata*. In contrast to As, Sb(III) is not translocated in large amounts to the shoots of this As hyperaccumulator species [208]. However, for *Pteris cretica*, similar shoot accumulation values for As and Sb have been reported [209].

Different systems may account for the transport of the pentavalent anionic antimoniate and arsenate [210]. In contrast to As(V), Sb(V) is apparently not taken up by phosphate transport systems. The mechanisms of SbV transport are still unclear.

## 5. Conclusions and Outlook

Trivalent inorganic cations can severely interfere with plant cell membranes. Fortunately, in most soil environments, the bioavailability of inorganic trivalent cations is rather low and membrane permeability to trivalent cations is reduced. However, plants have evolved different mechanisms to acquire and safely handle elements with a +3-oxidation state within a certain concentration range, which is species dependent. Figure 3 summarizes the main transporters involved in the loading, efflux, and storage of these elements. Redox active, essential metal ions, like Fe and Mn, with a functionally relevant +3-oxidation state in plant metabolism are taken up either as divalent cations or, in the case of Fe(III), in an organically complexed, uncharged form through YSL transporters. Metalloids or non metals in the +3-oxidation state like B(III), As(III), and Sb(III) can also enter plant cells in a neutral, hydroxylated form as B(OH)_3_, As(OH)_3_, or Sb(OH)_3_ using transporters of the aquaporin family. In contrast to the soluble hydroxyls of these elements, Al(OH)_3_ is insoluble and unavailable for membrane transport. Aluminum may also cross the plasma membrane in a neutral, soluble form as Al-malate by a NIP channel. Soluble monomeric Al, such as Al(OH)_2_^+^ or Al(OH)_2_^+^, may represent other possible Al forms available for membrane transport. Direct experimental evidence for this hypothesis is still missing.

In recent years, genomic analyses, homology modelling, heterologous expression of transporter proteins in yeast, and research with specific transporter mutants (mainly of *Arabidopsis* and rice) have allowed the identification and characterization of many new ion transporters and NIP channels in plants. In the case of trivalent elements, mechanisms of mobilization in the rhizosphere, binding to organic or inorganic ligands, and eventually redox reactions prior to root membrane transport, are essential features. Challenges for the future include better characterization of the differential transporter systems and transported chemical species at the tissular and subcellular level, in combination with a better understanding of the processes governing metal and metalloid binding and ligand exchanges during both subcellular distribution and long-distance transport from roots to leaves and to flowers and seeds. The identification of transporters responsible for the safe subcellular compartmentation of organic–metal complexes also requires further attention.

## Figures and Tables

**Table 1:** Standard electrode potential (E°) of elements with an unstable +3-oxidation state. Value for Ni ref. [48]; all others values from [49].

Element	Standard Electrode Potential E° (V)
Ni(OH)_3_/Ni(OH)_2_	+1.31
Cu^3+^/Cu^2+^	+2.40
Co^3+^(aq)/Co^2+^(aq)	+1.92
Mn^3+^/Mn^2+^	+1.54
Fe^3+^_(aq)_/Fe^2+^_(aq)_	+0.77
Cr(VI)/Cr(III)	+1.36
Cr(III)/Cr(II)	−0.41

## Abbreviations

ABC	ATP Binding Cassette
ACR	Arsenical Resistance Protein
AHA	H^+^-ATPase
AQPs	Aquaporins
BOR	Boron Transporter
CAX	Calcium/Proton Exchanger
CCX	Cation/Calcium Exchanger
CDF/MTP	Cation Diffusion Facilitator/Metal Tolerance Protein
CME	clathrin-mediated endocytosis
COPT	Copper Transporter
DMAs	dimethylarsenate
FPN	Ferroportin
FRD	Ferric Reductase Defective
FRO	Ferric Reductase Oxidase
HMA	Heavy Metal Associated Domain
IRT	Iron-Regulated Transporter
LSI	Low Silicon
MA	mugineic acid
Mas	methylarsenate
MOCO	Molybdenum Cofactor
MOT	Molybdenum Transporter
NA	nicotianamine
NIP	Nodulin26-like Intrinsic Protein
NRAMP	Natural Resistance Associated Macrophage Protein
NRAT	NRAMP Aluminum Transporter 1
ROS	reactive oxygen species
SLAC	Slow Anion Channel-associated
SOD	Superoxide Dismutase
SULT	Sulphate Transporter
VIT	Vacuolar Iron Transporter
YSL	Yellow Stripe-Like
ZIP	Zinc-regulated transporters, Iron-regulated transporter-like Protein
